# THE EFFECTS OF ICT USE ON PERSONAL MASTERY: RETIREMENT STATUS MATTERS

**DOI:** 10.1093/geroni/igad104.1064

**Published:** 2023-12-21

**Authors:** Alvin Ho, Daisy Li, Dannii Yeung

**Affiliations:** City University of Hong Kong, Hong Kong, Hong Kong; City University of Hong Kong, Hong Kong, Hong Kong; City University of Hong Kong, Hong Kong, Hong Kong

## Abstract

Internet and technology use have provided middle-aged or older adults with a new arena to regain control over their personal and social lives (McMellon & Schiffman, 2002) and improve their well-being (e.g., Sala et al., 2020; Shufford et al., 2021; Yeung et al., 2022). Still, whether such effects depend on retirement status has not been thoroughly examined. A total of 2391 middle-aged and older participants provided their retirement status (retired or not), and their use of information and communication technology (ICT) for different purposes (social, leisure, financial, and medical) and level of personal mastery were assessed. Four regression analyses were performed to test the moderating effect of retirement status on the relationship between each type of ICT use and personal mastery. Significant interaction was found between retirement and ICT use for leisure (β = .002, p = .046), financial (β = .003, p = .009), and, marginally, medical purposes (β = .002, p = .059), but not for social purpose (β = .000, p = .520). The beneficial effect of the use of ICT on personal mastery was shown to be either exclusive (for medical purpose) or significantly stronger (for leisure and financial purposes) for participants who were retired. This suggests that encouraging middle-aged and older adults to perform ICT-related activities may be particularly crucial when they are retired, presumably due to their capability to compensate for the loss of work-related control and identity.

